# Biological effects of air pollution on the function of human skin equivalents

**DOI:** 10.1096/fba.2023-00068

**Published:** 2023-10-03

**Authors:** Wil J. Reynolds, Ndubuisi Eje, Paul Christensen, Wen‐Hwa Li, Susan M. Daly, Ramine Parsa, Bhaven Chavan, Mark A. Birch‐Machin

**Affiliations:** ^1^ Dermatological Sciences, Institute of Translational and Clinical Research Newcastle University Newcastle upon Tyne UK; ^2^ Bedson Building, Newcastle University Newcastle upon Tyne UK; ^3^ Johnson and Johnson Consumer Inc. Skillman New Jersey USA; ^4^ Croda Europe Ltd Snaith UK

**Keywords:** aging, in vitro, ozone, particulate matter, pollution, skin

## Abstract

The World Health Organization reports that 99% of the global population are exposed to pollution levels higher than the recommended air quality guidelines. Pollution‐induced changes in the skin have begun to surface; however, the effects require further investigation so that effective protective strategies can be developed. This study aimed to investigate some of the aging‐associated effects caused by ozone and particulate matter (PM) on human skin equivalents. Full‐thickness skin equivalents were exposed to 0.01 μg/μL PM, 0.05 μg/μL PM, 0.3 ppm ozone, or a combination of 0.01 μg/μL PM and 0.3 ppm ozone, before skin equivalents and culture medium were harvested for histological/immunohistochemical staining, gene and protein expression analysis using qPCR, Western blotting, and ELISA. Markers include MMP‐1, MMP‐3, *COL1A1*, collagen‐I, 4‐HNE, HMGCR, and PGE2. PM was observed to induce a decrease in epidermal thickness and an enhanced matrix building phenotype, with increases in *COL1A1* and an increase in collagen‐I protein expression. By contrast, ozone induced an increase in epidermal thickness and was found to induce a matrix‐degrading phenotype, with decreases in collagen‐I gene/protein expression and increases in MMP‐1 and MMP‐3 gene/protein expression. Ozone was also found to induce changes in lipid homeostasis and inflammation induction. Some synergistic damage was also observed when combining ozone and 0.01 μg/μL PM. The results presented in this study identify distinct pollutant‐induced effects and show how pollutants may act synergistically to augment damage; given individuals are rarely only exposed to one pollutant type, exposure to multiple pollutant types should be considered to develop effective protective interventions.

## Graphical abstract

Skin equivalents were exposed to particulate matter and/or ozone before being assessed for gene and protein markers associated with dermal integrity, inflammation, and the skin barrier. Ozone induced significant increases in epidermal thickness, MMP expression and inflammatory marker PGE2. Particulate matter alone induced no significant changes; however, when combined with ozone synergistic effects were observed. These results identify distinct pollutant‐induced effects and demonstrate how pollutants may act synergistically to augment damage. This should be considered when developing effective protective interventions.

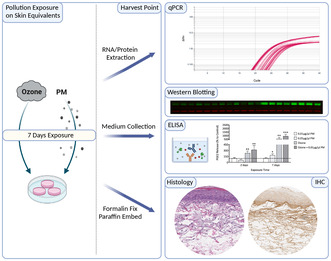

**Abbreviations**
4‐HNE: 4‐hydroxynonenal
BSA: bovine serum albumin
DMSO: dimethyl sulfoxide
ECM: extracellular matrix
HMGCR: 3‐hydroxy‐3‐methylglutaryl‐CoA reductase
MMP: matrix metalloproteinase
PAH: polycyclic aromatic hydrocarbons
PBS: phosphate buffered saline
PCR: polymerase chain reaction
PGE2: prostaglandin‐E2
PM: particulate matter
SD: standard deviation
TBST: tris buffered saline +0.2% tween‐20

## 1 INTRODUCTION

As the global population increases, the demand for further urbanization and industrialization also increases, leading to the increased production of ambient air pollutants which can be split into primary and secondary pollutants. Primary pollutants can be further subdivided into particulate matter (PM) and gases (CO, CO_2_, NO, NO_2_, NO_X_, and SO_2_), whereas secondary pollutants refer to those formed from reactions with primary pollutants, for example, ozone which is formed via photochemical reactions between primary pollutants, heat, and ultraviolet (UV) radiation.^1^ This ambient air pollution has been implicated in 4.2 million deaths annually, and although the majority of these deaths have been linked to cardiorespiratory conditions, the involvement of pollutants in the etiology of conditions involving other organs is now being considered.^2^ One such organ being considered due to its constant exposure to the environment is the skin, with a number of pollutants already being implicated in the detriment of skin structure and function. The pollutants that have been the focus of research in this area due to their environmental abundance and their potential to be modified by human activity are PM and ozone.^3^ Ambient PM represents a global health risk, being implicated in various cardiovascular and pulmonary diseases, as well as cancers, with the smaller particle sizes being considered more dangerous due to their ability to travel deeper into internal systems. It is only recently that the role of PM in the development of skin conditions and skin aging characteristics has been questioned, with a number of studies indicating a correlation between air pollution levels and the increased onset of skin conditions and/or skin aging characteristics.^4^, ^5^, ^6^ One such study observed a 20% increase in pigment spots on the cheeks and forehead with increased traffic‐associated PM exposure, while another study showed smokers were almost five times more likely to have facial wrinkles than nonsmokers.^7^, ^8^ It has been proposed that PM can exert effects through two means; the first is through the actual particle itself, with their large surface area rendering them highly reactive toward biological surfaces (such as skin).^9^ The second is their ability to act as carriers for organic compounds such as polycyclic aromatic hydrocarbons (PAHs), which are capable of adsorbing on to the surface of PM and exerting their own effects.^10^ These effects are not limited to the parent compound of PAHs but also from photo‐modified products which have distinct bioactivities in comparison to the parent compound. The aromatic rings of PAHs are able to absorb solar light in both the visible and UV spectrum, allowing PAHs to undergo photo‐oxidation and transform into ROS‐forming quinones; some evidence indicates that with an increased number of aromatic rings comes an increased potency of phototoxicity.^11^ Ozone is a gaseous secondary pollutant present in both the Earth's upper atmosphere (stratosphere), and also at ground level (troposphere) where it is found at high concentrations in smog. Tropospheric ozone, or “bad ozone”, is mainly formed through the chemical reactions between nitrogen oxides and volatile organic compounds (VOC) in the presence of sunlight.^12^ These compounds come from various sources; however, around 50% of nitrogen oxides and 45% of VOCs originate from vehicle emissions, with the remaining percentage originating from industrial processes such as power plants and oil refinery processes.^13^ Alongside PM, ozone exposure has also been implicated in the decline of skin health, with a higher incidence of hospital admissions for skin related conditions being observed in populations exposed to higher levels of ozone, and further studies showing ozone‐induced lipid peroxidation, inflammation, and structural damage.^14^, ^15^

Environmental stressors have the ability to induce differential effects in the epidermis and dermis, with epidermal cells showing the ability to adapt in order to protect against further damage, and dermal cells modulating characteristic signs of skin aging. Keratinocytes in the epidermal layer have the ability to adapt to different extrinsic stimuli, with damage inducing the activation of genes like p53 which can regulate cell cycle arrest, DNA repair pathways, and apoptosis. After damage has subsided, keratinocytes proliferate rapidly, and the increased epidermal thickness allows for superior protection against UV penetration.^16^ Their involvement in the formation of the stratum corneum also provides the body with a protective barrier against environmental assaults, preventing penetration of a wide range of chemicals and microbes. Alongside the corneocytes, the lipid envelope provides the primary barrier to the transcutaneous movement of water and electrolytes, and consists of ceramides, cholesterol, and free fatty acid. Upon acute barrier disruption, enzymes involved in epidermal cholesterol synthesis (such as HMG‐CoA reductase) are rapidly upregulated to restore the lipid barrier and maintain protection against external stressors and transepidermal water loss.^17^ These lipids can also undergo peroxidation after exposure to stressors that induce oxidative stress, and the newly formed peroxidation products can then act as second messengers in the induction of downstream processes, such as the activation of NF‐κB and AP‐1 transcription factors that can promote the expression of proinflammatory cytokines and matrix metalloproteinase (MMPs).^18^ Environmental stressors therefore have the ability to induce different extracellular matrix (ECM) profiles via dysregulation of ECM degrading proteins such as MMPs, causing disruption to ECM proteins such as collagen and consequently skin integrity.^19^ This change also occurs with age, as skin cells undergo a genetic switch in expression patterns, transferring from matrix secreting to a matrix‐degrading phenotype, this is thought to be in part due to the induction of low‐level chronic inflammation termed “inflammaging”.^20^

Given the recent findings on pollutant‐induced effects on the skin, it was of interest to not only further investigate the different biological effects of PM and ozone on the skin, but also how these pollutants may interact to cause synergistic damage.

## 2 MATERIALS AND METHODS

### 2.1 Skin equivalent culture

Phenion full‐thickness skin equivalents (Henkel AG & Co. KGaA, Düsseldorf) were cultured in a separate culture setup as per manufacturer's guidelines.

### 2.2 Pollutant exposure

PM (Urban Dust 1649b, NIST) was prepared in phosphate buffered saline (PBS) to a concentration of 0.01 μg/μL or 0.05 μg/μL, and sonicated in a sonic bath for 1 h with vortexing every 10 min to avoid agglomeration, before applying in a 25 μL volume to the surface of each skin equivalent. Ozone was generated and passed through an exposure chamber containing skin equivalents using nitrogen gas as a carrier. Ozone generation characterization and chamber construction was performed under the supervision of Professor Paul Christensen, with the help of Ndubuisi Eje. Skin equivalents were exposed to either 0.01 μg/μL PM, 0.05 μg/μL PM, 0.3 ppm ozone, or a combination of 0.3 ppm ozone and 0.01 μg/μL PM. The untreated control group consisted of equivalents being exposed to 0.2% DMSO in PBS. After the 8‐h exposure period, all skin equivalents were washed with PBS eight times, returned to the incubator overnight and this process was repeated for 7 days.

### 2.3 RNA extraction

Skin equivalents were homogenized in Precellys CKMix50 R tubes containing prechilled RLT buffer (with freshly supplemented β‐mercaptoethanol) in a Precellys Evolution tissue homogenizer (Bertin Instruments, UK), with the following settings implemented: 6000 rpm for 60 s at 0°C, followed by a 30 s pause, before repeating this process for a further 5 cycles. The tubes were centrifuged at 12,000 rpm for 5 min at 4°C before transferring the supernatant to prechilled RNase‐free microcentrifuge tubes. The Qiagen RNeasy mini kit was used for the remainder of the RNA extraction, using the purification of total RNA from animal tissue with optional on column DNase digestion protocol.

### 2.4 Reverse transcription of RNA

RNA was used as a template to produce cDNA via reverse transcription in order to analyze relative expression of a gene transcript using quantitative PCR. Reverse transcription was performed with the High‐Capacity cDNA Reverse Transcription Kit (Applied Biosystems, UK) using the GeneAmp PCR System 9700 (Applied Biosystems, UK) as per manufacturer's guidelines.

### 2.5 Quantitative PCR

Quantitative PCR (qPCR) was performed on cDNA to analyze any changes in the expression levels of genes associated with skin wrinkling and lipid synthesis. qPCR was performed with TaqMan Fast Advanced Master Mix and 40 ng cDNA using the QuantStudio 3 Real‐Time PCR System. Primers were purchased from Thermo Fisher Scientific; *GAPDH* (Hs02786624_g1), *MMP‐1* (Hs00899658_m1), *MMP‐3* (Hs00968305_m1), *HMGCR* (Hs00168352_m1), and *COL1A1* (Hs00164004_m1).

### 2.6 Enzyme‐linked immunosorbent assay

Culture medium was harvested after 2 and 7 days of pollution exposure, centrifuged at 200 × *g* for 5 min at 4°C before removing the supernatant and analyzing PGE2 secretion using ELISA (Thermo Fisher Scientific, UK) following the manufacturer's guidelines. Background absorbance using blank culture medium was subtracted from all datapoints, before using a 4‐parameter algorithm to create a standard curve and interpolating unknown sample PGE2 concentrations.

### 2.7 Histological analysis

Skin equivalents were placed between two biopsy foam pads which were then sandwiched in a tissue embedding cassette and placed in a Histotainer II specimen container prefilled with 10% neutral‐buffered formalin (Sigma Aldrich, UK). Skin equivalent paraffin embedding, processing, sectioning, and H&E staining were performed by NovoPath (Royal Victoria Infirmary, Newcastle upon Tyne). The Leica SCN400 Brightfield Slide Scanner and Autoloader system was then used to acquire full section images of stained slides. Epidermal thickness measurements were conducted using QuPath, with around 150–350 individual measurements taken for each skin equivalent.

### 2.8 Western blotting

Skin equivalents were homogenized in Precellys CKMix50 R tubes containing prechilled RIPA buffer (with freshly supplemented protease inhibitor cocktail) in a Precellys Evolution tissue homogenizer (Bertin Instruments, UK), with the following settings implemented: 6000 rpm for 60 s at 0°C, followed by a 30 s pause, before repeating this process for a further 5 cycles. Homogenates were incubated on ice for 5 min before centrifuging at 12,000 rpm for 20 min at 4°C, before using the supernatant for protein analysis. Bradford assay was used to quantify protein concentration, and samples were prepared with NuPAGE Sample Reducing Agent (Invitrogen, UK) and NuPAGE LDS Sample Buffer (Invitrogen, UK) before heating at 70°C for 10 min. SDS‐PAGE was performed, and proteins were then electro‐transferred on to the nitrocellulose membrane using the iBlot 2 semidry transfer system (Invitrogen, UK). Immunodetection was subsequently performed using MMP‐1 (R&D Systems, 1/1000), MMP‐3 (R&D Systems, 1/1000), GAPDH (Santa Cruz, 1/2000), HMGCR (Novus Biologicals, 1/1000), and appropriate IRDye 800CW and IRDye 680RD secondary antibodies. Membranes were then imaged using the LI‐COR Fc Dual Mode Imaging System. Densitometric analysis was then performed to analyze protein expression.

### 2.9 Immunohistochemistry

Skin equivalent sections (4 μm) were incubated at 37°C for 1 day prior to staining to allow maximum tissue adherence to the slide. Sections were deparaffinized by incubating at 60°C, and taking through xylenes, before rehydrating sections through graded ethanols. Citrate buffer antigen retrieval was performed before continuing with either fluorescent or chromogenic immunohistochemistry. For fluorescent immunohistochemistry sections were blocked with blocking buffer (5% goat serum, 0.2% v/v Triton‐X100 in TBS) for 1 h before incubation with primary antibody (R&D Systems, 1/1000) in antibody incubation buffer (1% BSA and 0.2% v/v Triton‐X100 in TBS) overnight at 4°C. Sections were washed and incubated with secondary antibody in antibody incubation buffer for 1 h at room temperature in the dark. Sections were finally incubated with 0.1 μg/mL 4′,6‐diamidino‐2‐phenylindole (DAPI) for 15 min at room temperature before mounting coverslips with Prolong Antifade Glass. Chromogenic immunohistochemistry was conducted using the ImmPRESS HRP Universal PLUS Polymer Kit (Vector Labs, USA) to assess collagen‐I expression (Abcam, 1/500). Manufacturer's guidelines were followed, with the following amendments; (a) normal horse serum blocking time extended from 30 min to 1 h, (b) ImmPRESS HRP Universal PLUS Polymer incubation time extended to 1 h, and (c) washing steps increased to three washes. Once brown color development was observed, the reaction was stopped, before cell nuclei were counterstained with hematoxylin, differentiated with 1% v/v acid alcohol and the nuclei blued in ammonia water. Sections were dehydrated in graded ethanols, before clearing in two rounds of xylene, allowing to dry and mounting coverslips using DPX mountant.

### 2.10 Statistical analyses

Statistical analysis was conducted using GraphPad Prism 9. Percentage change to untreated control data and qPCR results (calculated using 2^−ΔΔCt^ method) were first logged as fractions before performing one sample *t*‐tests to assess whether average percentage changes differ significantly from the hypothetical logged mean of the untreated control. Unpaired *t*‐tests were used to assess synergism between the ozone and ozone +0.01 μg/μL PM exposure groups. Results are presented as mean with standard deviation (SD) represented by the error bars, with the dashed line representing the mean of the untreated control.

## 3 RESULTS

### 3.1 Effects of pollutants on skin equivalent integrity

**FIGURE 1:**
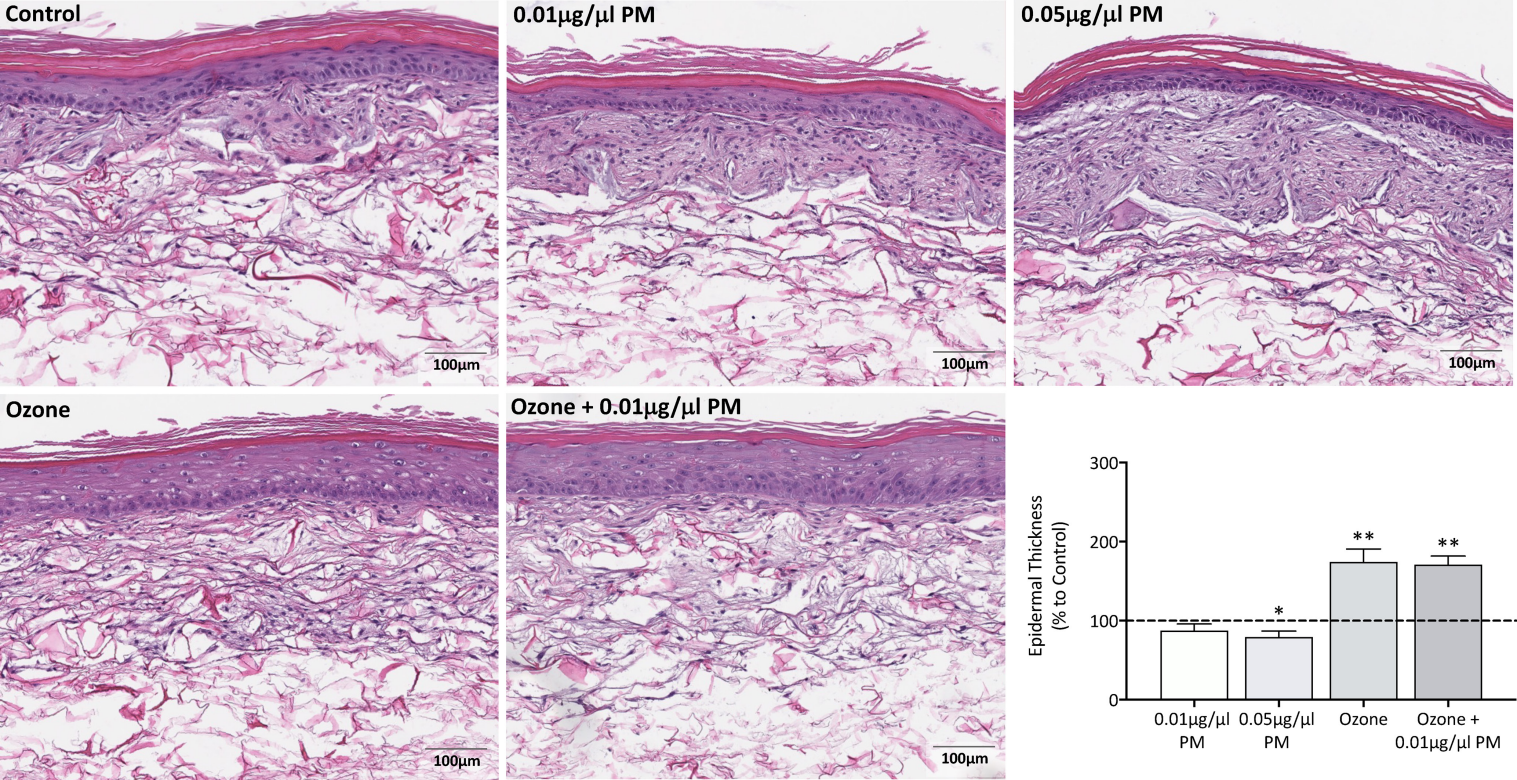
Hematoxylin and eosin (H&E) staining and analysis of viable epidermal thickness of Phenion FT skin equivalents after single and combination pollutant exposure for 7 days. Phenion FT skin equivalents were exposed to either 0.2% DMSO in PBS (untreated control), 0.01 μg/μL PM, 0.05 μg/μL PM, 0.3 ppm ozone, or a combination of 0.01 μg/μL PM and 0.3 ppm ozone for 8 h daily for a total of 7 days. After 7 days, skin equivalents were harvested, fixed in 10% neutral‐buffered formalin, paraffin embedded, and processed before H&E staining. Full H&E stained Phenion FT skin equivalent sections were scanned using a Leica SCN400 Brightfield Slide Scanner at 40×, with a representative image for each skin equivalent presented. Scale bars: 100 μm. Viable epidermal thickness (stratum basale to the stratum granulosum) for the entire skin equivalent was measured using QuPath with around 300–400 individual measurements obtained for each equivalent, before averaging and presenting as mean percentage change to the untreated control+SD. One sample *t*‐test was performed, with *p < 0.05 and **p < 0.01 representing significance when compared to the untreated control and an unpaired *t*‐test was performed to assess synergism between ozone and ozone +0.01 μg/μL PM, *n* = 4.

Upon visual assessment, it was noted that PM seemed to alter stratum corneum structure to the point of reducing the viable epidermal thickness, indicating an increase in keratinocyte turnover rate (Figure 1). This is more apparent in the higher 0.05 μg/μL PM group, where the stratum corneum lacks typical structure, with no compact layer and an increased basket weave layer. On the contrary, ozone and a combination of ozone and 0.01 μg/μL PM‐induced thickening of the viable epidermal layer. Analysis of viable epidermal thickness confirmed visual assessment of pollutant‐induced changes. Viable epidermal thickness was decreased by 12 (*p* = 0.0602) and 19.9% (*p* = 0.0179) after exposure to 0.01 μg/μL and 0.05 μg/μL PM, respectively. Ozone induced an increase of 74.8% (*p* = 0.0013) when applied alone, and an increase of 71.6% (*p* = 0.0045) when applied in combination with 0.01 μg/μL PM. There were no synergistic changes in viable epidermal thickness when comparing ozone to ozone +0.01 μg/μL PM.

### 3.2 Effects of pollutants on the collagen synthesis pathway

**FIGURE 2:**
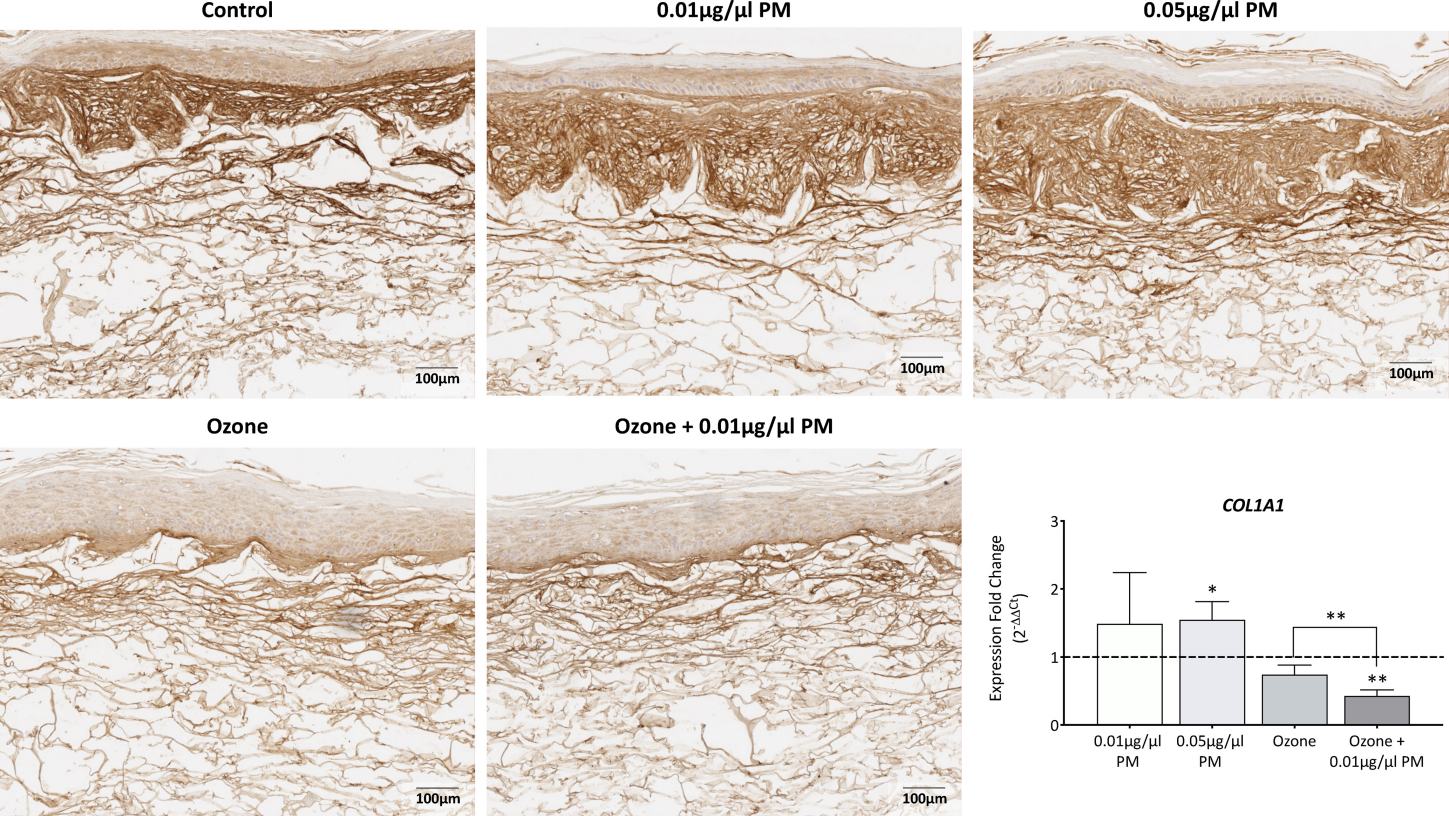
Analysis of collagen‐I gene and protein expression in Phenion FT skin equivalents after single and combination pollutant exposure for 7 days. Phenion FT skin equivalents were exposed to either 0.2% DMSO in PBS (untreated control), 0.01 μg/μL PM, 0.05 μg/μL PM, 0.3 ppm ozone, or a combination of 0.01 μg/μL PM and 0.3 ppm ozone for 8 h daily for a total of 7 days before extracting RNA protein. RNA was reverse transcribed into cDNA and TaqMan gene expression analysis was then used to analyze changes in *COL1A1* gene expression. Relative changes were assessed using the 2^−ΔΔCt^ method and presented as fold change to the untreated control+SD, with the dashed line representing the mean untreated control expression levels. Immunohistochemical staining for collagen‐I protein expression in formalin‐fixed paraffin‐embedded skin equivalents was also performed, with cell nuclei counterstained with hematoxylin. Scale bars: 100 μm. One sample *t*‐test was performed, with *p < 0.05 and **p < 0.01 representing significance when compared to the untreated control and an unpaired *t*‐test was performed to assess synergism between ozone and ozone +0.01 μg/μL PM.

Distinct effects in *COL1A1* gene expression were observed with different pollutant types, with PM inducing an increase and ozone inducing a decrease in expression (Figure 2). PM induced an upregulation of 1.49 ± 0.75 (*p* = 0.2791) and 1.55 ± 0.27 (*p* = 0.021) for 0.01 μg/μL and 0.05 μg/μL PM, respectively. By contrast, ozone induced a downregulation of 0.74 ± 0.14 (*p* = 0.0569), and a significant downregulation of 0.43 ± 0.09 (*p* = 0.0031) when in combination with 0.01 μg/μL PM. A significant synergistic downregulation was observed when comparing ozone to a combination of ozone + 0.01 μg/μL PM (*p* = 0.009).

Qualitative immunohistochemical assessment of collagen‐I protein expression seemed to mirror that of *COL1A1* gene expression analysis (Figure 2). Exposure to PM seemed to result in an increase in collagen staining, specifically within the papillary dermis which seemed to increase with increasing PM concentration. By contrast, ozone and a combination of ozone +0.01 μg/μL PM seemed to cause a decrease in collagen‐I staining, with a diminished papillary dermis and thinner collagen fibers observed in the reticular dermis. No nonspecific binding was observed in the secondary control (Figure S1A).

### 3.3 Effects of pollutants on ECM remodeling

**FIGURE 3:**
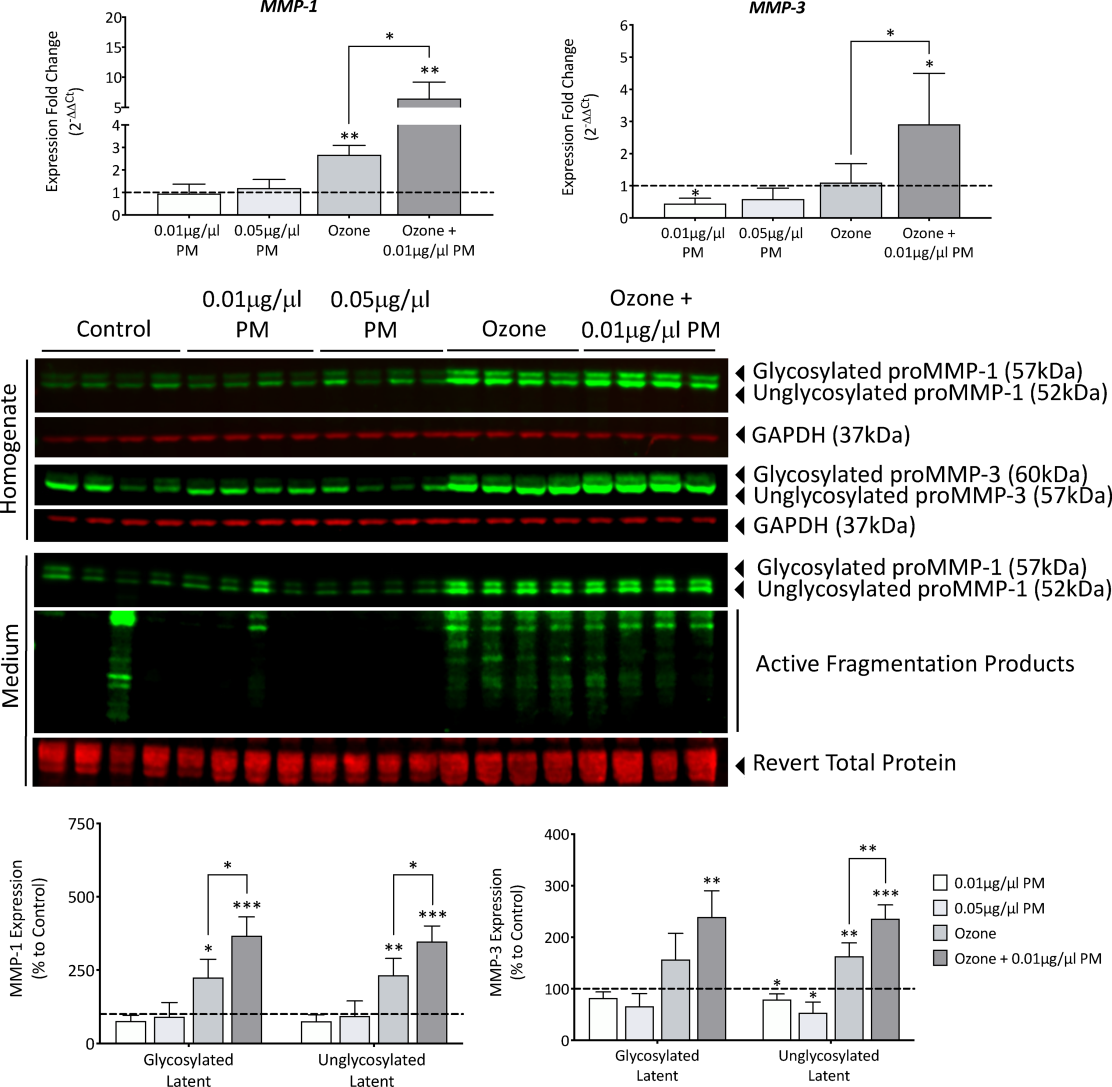
Analysis of MMP‐1 and MMP‐3 gene and protein expression in Phenion FT skin equivalents after single and combination pollutant exposure for 7 days. Phenion FT skin equivalents were exposed to either 0.2% DMSO in PBS (untreated control), 0.01 μg/μL PM, 0.05 μg/μL PM, 0.3 ppm ozone, or a combination of 0.01 μg/μL PM and 0.3 ppm ozone for 8 h daily for a total of 7 days before homogenizing and extracting RNA and protein. RNA was reverse transcribed into cDNA and TaqMan gene expression analysis was then used to analyze changes in *MMP‐1* and *MMP‐3* gene expression and relative changes were assessed using the 2^−ΔΔCt^ method and presented as fold change to the untreated control+SD, with the dashed line representing the mean untreated control expression levels. Western blotting was then used to analyze changes in MMP‐1 and MMP‐3 protein expression, with expression normalized to GAPDH using densitometric analysis, before presenting as mean percentage change to the untreated control+SD. Culture medium was also collected after 7 days of exposure, concentrated and Western blotting was used to analyze extracellular MMP‐1 expression, normalized to total protein revert stain. One sample *t*‐test was performed, with *p < 0.05, **p < 0.01 and ***p < 0.001 representing significance when compared to the untreated control and an unpaired *t*‐test was performed to assess synergism between ozone and ozone +0.01 μg/μL PM, *n* = 4.

Neither 0.01 μg/μL (0.96 ± 0.42), nor 0.05 μg/μL (1.20 ± 0.37) PM exposure induced any significant changes in *MMP‐1* gene expression (Figure 3). Similar to *COL1A1*, ozone seemed to be the main driver of damage, with ozone (2.67 ± 0.42, *p* = 0.0011) and a combination of ozone and 0.01 μg/μL (6.46 ± 2.72, *p* = 0.0053) causing significant increases in *MMP‐1* gene expression. A synergistic effect was also observed when comparing single ozone exposure to a combination of ozone +0.01 μg/μL PM (*p* = 0.02). *MMP‐3* gene expression showed the same pattern of induction as *MMP‐1* however with a lower level of induction (Figure 3). Exposure to 0.01 μg/μL PM (0.45 ± 0.42, *p* = 0.018) and 0.05 μg/μL PM (0.59 ± 0.33, *p* = 0.1041) induced downregulation in *MMP‐3* gene expression. Ozone induced no significant changes in expression when applied alone; however, when in combination with 0.01 μg/μL PM induced an upregulation of (2.90 ± 1.59, *p* = 0.0339). A synergistic effect was also observed when comparing single ozone exposure to a combination of ozone +0.01 μg/μL PM (*p* = 0.04).

Western blotting was used to assess pollutant‐induced changes in the protein expression of proMMP‐1 and proMMP‐3 in skin equivalent homogenates. MMP‐1 protein expression followed the same pattern as that of the *MMP‐1* gene expression, with PM alone causing no changes, and ozone causing increases of 125.2% (*p* = 0.0159) in glycosylated proMMP‐1 and 133% (*p* = 0.0082) in unglycosylated proMMP‐1 (Figure 3). When ozone and 0.01 μg/μL PM were applied in combination, both glycosylated (267.4%, *p* = <0.001) and unglycosylated (247.7%, *p* = <0.001) proMMP‐1 protein expression increased more dramatically than ozone exposure alone. A significant synergistic effect was in fact observed for both glycosylated (*p* = 0.03) and unglycosylated (*p* = 0.03) proMMP‐1 expression when comparing single ozone exposure to a combination of ozone +0.01 μg/μL PM. No pollutant induced‐MMP‐1 activation was noted in skin equivalent homogenate. MMP‐3 protein expression followed a similar pattern to that of the *MMP‐3* gene expression, with PM alone causing downregulation, and a combination of ozone and 0.01 μg/μL PM causing an upregulation in protein expression, again showing lower levels of induction (Figure 3). However, despite no change in *MMP‐3* gene expression, MMP‐3 protein expression was upregulated after ozone exposure. Exposure to 0.01 μg/μL PM‐induced downregulations of 17.8 (*p* = 0.0694) in glycosylated proMMP‐3 and 20.7 (*p* = 0.0412) in unglycosylated proMMP‐3, with similar downregulations of 34% (*p* = 0.0736) in glycosylated proMMP‐3 and 46.4% (*p* = 0.046) in unglycosylated proMMP‐3 after exposure to 0.05 μg/μL PM. Ozone caused an increase in both glycosylated (56.8%, *p* = 0.0815) and unglycosylated (63.3%, *p* = 0.0085) proMMP‐3 expression. When ozone and 0.01 μg/μL PM were applied in combination, both glycosylated (139.6%, *p* = 0.0039) and unglycosylated (136.2%, *p* = <0.001) proMMP‐3 protein expression increased more dramatically. A significant synergistic effect was in fact observed for unglycosylated (*p* = 0.008) proMMP‐3 and a trending significance for glycosylated proMMP‐3 (*p* = 0.06) when comparing single ozone exposure to a combination of ozone and 0.01 μg/μL PM. Increasing membrane exposure revealed that both ozone and a combination of ozone and 0.01 μg/μL PM seemed to induce activation‐associated fragmentation of proMMP‐3, showing both the 45 kDa activeMMP‐3 form, in addition to multiple lower molecular weight fragments associated with activation (data not shown due to resulting overexposure of proMMP bands). In conditioned medium, MMP‐1 expression followed a similar pattern, with ozone exposure inducing increases of 253.2 (*p* = 0.0021) and 197.4% (*p* = 0.0029) in glycosylated and unglycosylated proMMP‐1 expression, and a combination of ozone and 0.01 μg/μL PM inducing increases of 332.2 (*p* = 0.0135) and 218.4% (*p* = 0.0087) in glycosylated and unglycosylated proMMP‐1 (Figure 3). There was also increased activation‐associated fragmentation of proMMP‐1 after both ozone and combination pollution exposure in all biological replicates, with only one control replicate and one 0.05 μg/μL PM replicate showing any fragmentation.

### 3.4 Effects of pollutants on lipid homeostasis

**FIGURE 4:**
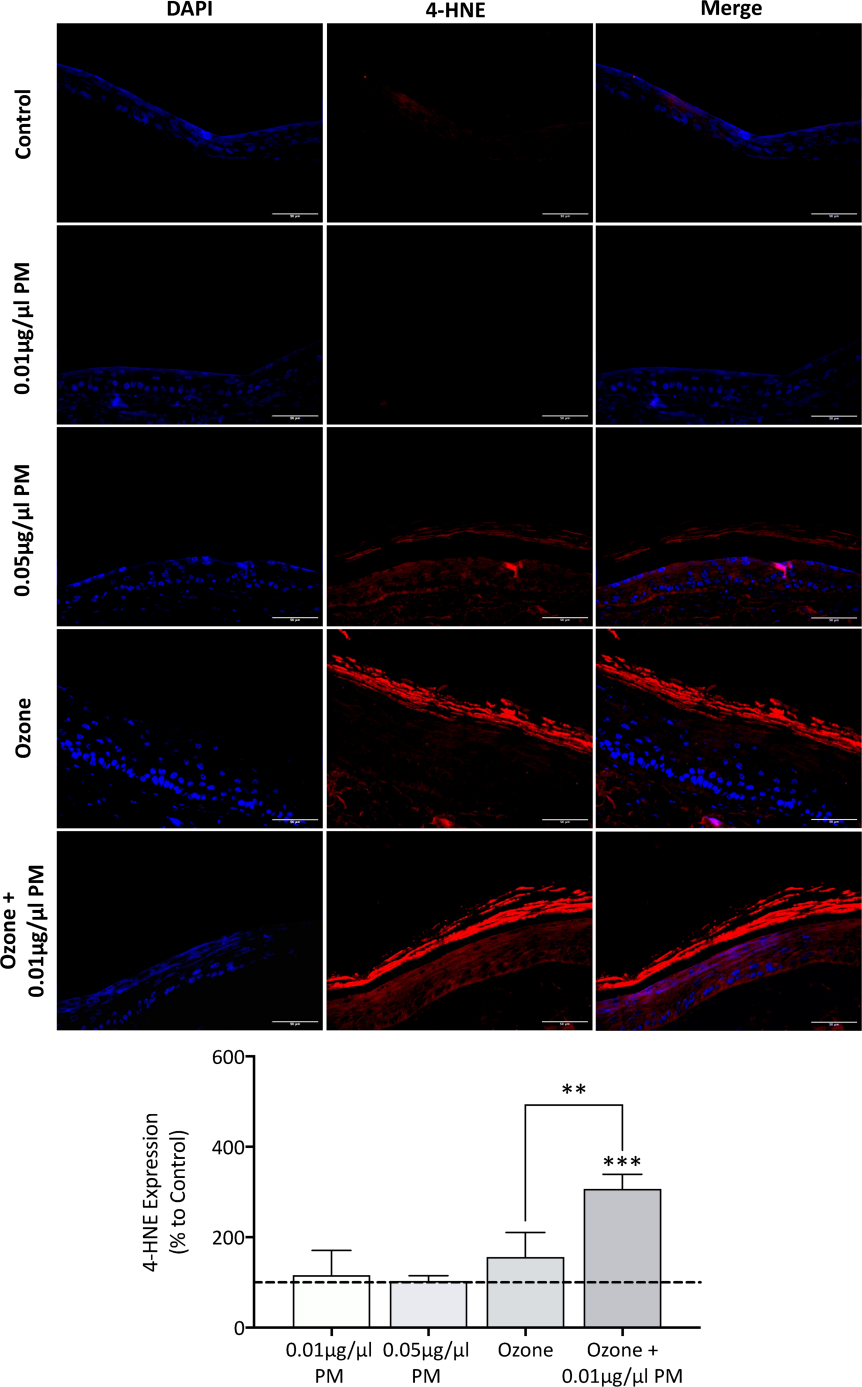
Immunohistochemical staining and analysis of Phenion FT skin equivalents for lipid peroxidation marker 4‐hydroxynonenal (4‐HNE) after single and combination pollutant exposure for 7 days. Phenion FT skin equivalents were exposed to either 0.2% DMSO in PBS (untreated control), 0.01 μg/μL PM, 0.05 μg/μL PM, 0.3 ppm ozone, or a combination of 0.01 μg/μL PM and 0.3 ppm ozone for 8 h daily for a total of 7 days before harvesting, fixing in 10% neutral‐buffered formalin, paraffin embedding, and immunohistochemical staining for 4‐HNE. Cell nuclei were counterstained with DAPI, and a representative image for each skin equivalent presented. Scale bars: 50 μm. Lipid peroxidation marker 4‐HNE staining was quantified in the stratum corneum of skin equivalents using Image J. Results are presented as the mean percentage change in mean fluorescent intensity to the untreated control+SD. Ten to twenty individual measurements for each skin equivalent were obtained for each exposure group, *n* = 4. One sample *t*‐test was performed, with **p < 0.01 and ***p < 0.001 representing significance when compared to the untreated control and an unpaired *t*‐test was performed to assess synergism between ozone and ozone +0.01 μg/μL PM.

Phenion FT skin equivalent sections were stained for the lipid peroxidation marker 4‐hydroxynonenal (4‐HNE) using fluorescent immunohistochemistry. Staining was present in both the stratum corneum and keratinocytes in the viable epidermal layer, however, because the stratum corneum is the first point of contact for pollutants and has a higher level of lipids, the staining within the stratum corneum was analyzed (Figure 4). The secondary control revealed some fluorescent staining within the reticular dermis, most likely attributed to autofluorescence of ECM proteins and would not affect analysis of the stratum corneum (Figure S1B). No changes in lipid peroxidation were observed after exposure to PM, however, an increase of 56.7% (*p* = 0.0792) after ozone exposure, and a further increase of 207% (*p* = <0.001) after exposure to a combination of ozone +0.01 μg/μL PM was observed. A significant synergistic effect in lipid peroxidation was observed when comparing ozone exposure to a combination of ozone +0.01 μg/μL PM (*p* = 0.005).

**FIGURE 5:**
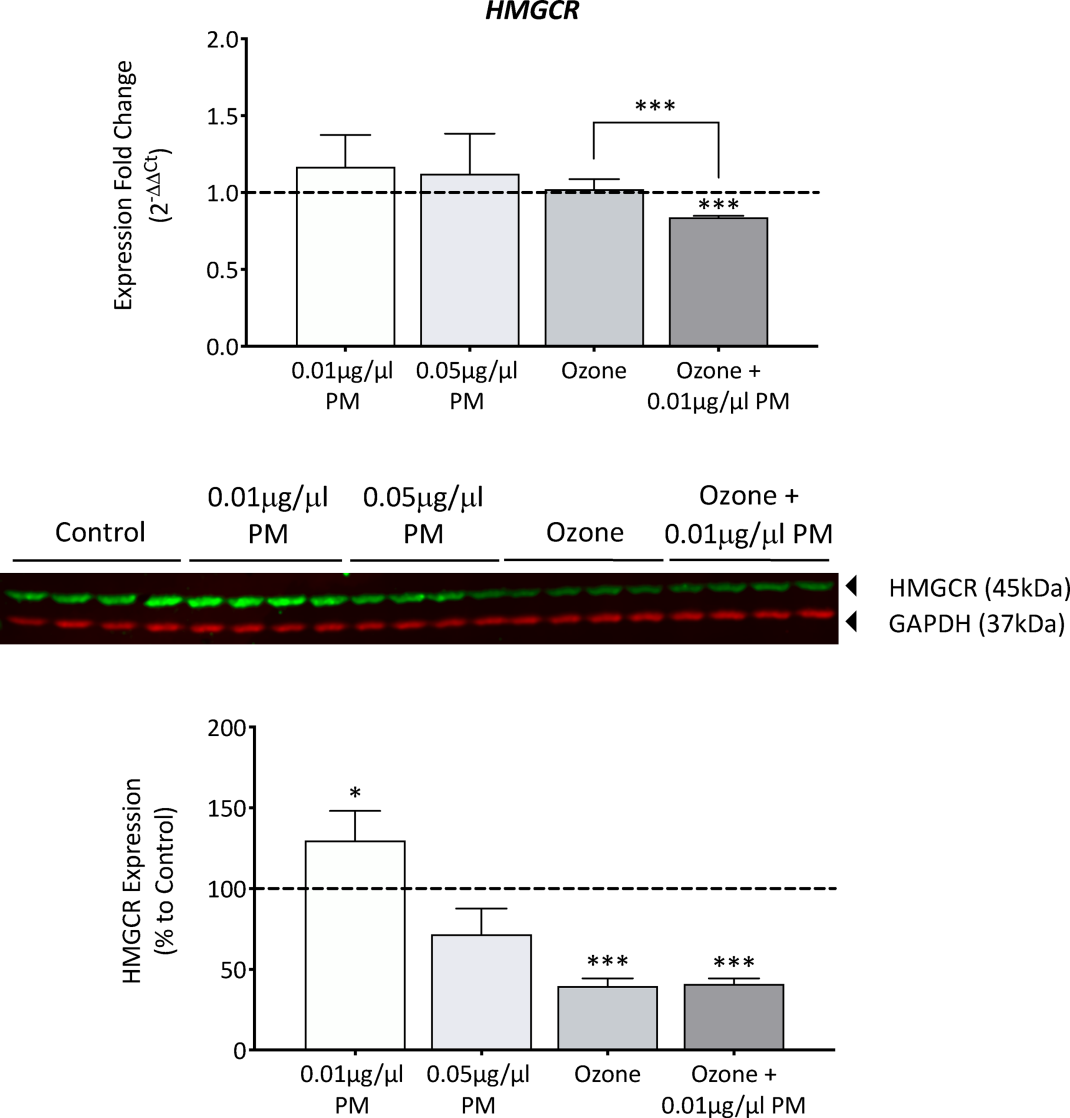
Analysis of HMGCR gene and protein expression in Phenion FT skin equivalents after single and combination pollutant exposure for 7 days. Phenion FT skin equivalents were exposed to either 0.2% DMSO in PBS (untreated control), 0.01 μg/μL PM, 0.05 μg/μL PM, 0.3 ppm ozone, or a combination of 0.01 μg/μL PM and 0.3 ppm ozone for 8 h daily for a total of 7 days before extracting RNA protein. RNA was reverse transcribed into cDNA and TaqMan gene expression analysis was then used to analyze changes in *HMGCR* gene expression. Relative changes were assessed using the 2^−ΔΔCt^ method and presented as fold change to the untreated control+SD, with the dashed line representing the mean untreated control expression levels. Western blotting was used to analyze changes in HMGCR protein expression, expression was normalized to GAPDH using densitometric analysis, before presenting as mean percentage change to the untreated control+SD. One sample *t*‐test was performed, with *p < 0.05 and ***p < 0.001 representing significance when compared to the untreated control and an unpaired *t*‐test was performed to assess synergism between ozone and ozone +0.01 μg/μL PM, *n* = 4.

*HMGCR* gene expression was also analyzed to note any changes in lipid synthesis status (Figure 5). While PM and ozone induced no significant changes in *HMGCR* gene expression individually, in combination there was a significant downregulation of 0.84 ± 0.009 (*p* = <0.001), and a significant synergistic effect when comparing ozone to a combination of ozone +0.01 μg/μL PM (*p* = <0.001). The protein expression of HMGCR showed distinct changes dependent on pollutant type (Figure 5). PM‐induced concentration‐dependent effects, with the lower PM concentration of 0.01 μg/μL upregulating HMGCR expression (+29.9%, *p* = 0.0334) whereas the higher concentration of 0.05 μg/μL downregulated expression (−28.3%, *p* = 0.0691). Ozone induced a downregulation of 60.2% (*p* = <0.001), and a similar downregulation of 59% (*p* = <0.001) when applied in combination with 0.01 μg/μL PM, with no significant synergistic effects observed.

### 3.5 Mediation of inflammation by pollutants

**FIGURE 6:**
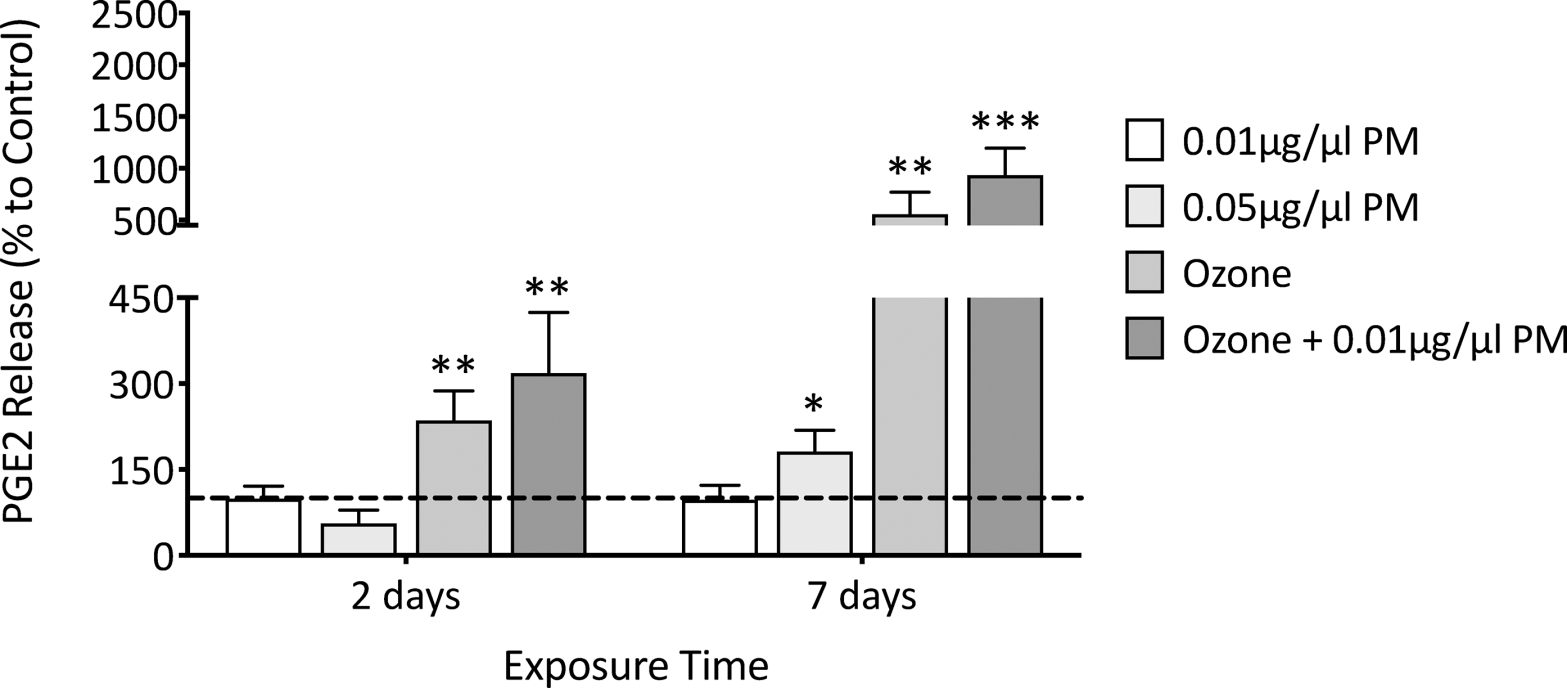
Analysis of PGE2 release in medium from Phenion FT skin equivalents after single and combination pollutant exposure. Phenion FT skin equivalents were exposed to 0.2% DMSO in PBS (untreated control), 0.01 μg/μL PM, 0.05 μg/μL PM, 0.3 ppm ozone, or a combination of 0.01 μg/μl PM and 0.3 ppm ozone for 8 h daily for a total of 7 days, with the medium removed after 2 and 7 days of exposure and analyzed for PGE2 release using ELISA. PGE2 was presented as mean percentage change to the untreated control+SD, with the dashed line representing untreated control mean, *n* = 4. One sample t‐test was performed, with *p < 0.05, **p < 0.01, and ***p < 0.001 representing significance when compared to the untreated control and an unpaired t‐test was performed to assess synergism between ozone and ozone +0.01 μg/μL PM.

Culture medium was harvested after both 2 and 7 days of pollutant exposure to assess PGE2 release to note both early‐ and late‐stage inflammatory responses (Figure 6). Exposure to 0.01 μg/μL PM induced no change in PGE2 release; however, exposure to 0.05 μg/μL PM induced an increase of 81.3% (*p* = 0.0112) after 7 days of exposure when compared to the untreated control. By contrast, ozone induced significant increases in PGE2 after both 2 (136%, *p* = 0.005) and 7 (456.4%, *p* = 0.0028) days of exposure, and when combined with 0.01 μg/μL PM induced larger increases after both 2 (218.5%, *p* = 0.0073) and 7 (834.3%, *p* = <0.001) days of exposure. While no significant synergistic effects were observed when comparing ozone to a combination of ozone +0.01 μg/μL PM, there were larger increases after both 2 (*p* = 0.23) and 7 (*p* = 0.07) days of exposure to a combination of ozone +0.01 μg/μl PM.

## 4 DISCUSSION

H&E staining showed ozone exposure induced an increase in cellular damage‐associated perinuclear vacuolisation of keratinocytes after ozone exposure. Ozone exposure was also shown to induce thickening of the viable epidermis, which is most likely a similar protective mechanism to that observed after UV damage subsides. The induction of cell damage and the resulting activation of damage response pathways subsequently leads to the increase in keratinocyte proliferation rate, allowing keratinocytes to not only replace those lost as a consequence of damage, but also to provide transient protection against stressors.^16^, ^21^ In contrast PM exposure induced a concentration‐dependent decrease in viable epidermal thickness and increase in the thickness of the upper stratum corneum. These characteristics are typical of a chloracne phenotype and can be attributed to the early onset of keratinocyte differentiation, this has been shown to be induced by 2,3,7,8‐Tetrachlorodibenzo‐*p‐*dioxin (TCDD)‐mediated AHR activation.^22^ TCDD is one of the components of the PM mixture used in this study, in addition to numerous other AHR ligands, this could explain the PM‐induced chloracne phenotype observed in this study. PM also affected stratum corneum structure, showing a concentration‐dependent decrease in the lower compact layer, and an increase in the thickness of the upper basket weave layer. The basket weave formation is a result of xylene or other clearing agents during histological processing extracting lipids between corneocytes due to the lack of corneodesmosomes in the upper layer; however, this layer does have a physiological role and incorrect formation may indicate dysregulation.^23^ The lipids formed in the intercellular spaces between corneocytes have been shown to have roles in maintaining skin softness and skin hydration by providing the skins water barrier and limiting any transepidermal water loss.^23^ The upper stratum corneum is formed by the controlled degradation of the adhering junctions between corneodesmosomes by proteases such as cathepsins and kallikreins.^24^ The increase in basket weave structure after PM exposure could be a result of a decreased corneodesmosomes formation or an increased degradation rate of corneodesmosomes; this could be plausible with research showing PM induces decreases in desmocollin‐1 (involved in corneodesmosomes structure), in addition to causing increases in a number of kallikreins.^25^, ^26^ PM exposure may also somehow increase lipid production, leading to a larger lipid volume between corneocytes and therefore an increased basket weave structure after histological processing. Ozone exposure also seemed to result in a reduced papillary dermis, with the characteristic thick collagen bundles of the reticular dermis extending into the diminished papillary dermis. This is an established phenomenon in skin aging, and studies specifically assessing papillary and reticular fibroblasts in skin development have theorized this may be due to a reduced sensitivity to mitogenic factors resulting in decreased growth potential of papillary fibroblasts.^27^, ^28^, ^29^

Pollutant‐induced changes were also observed at multiple points of the collagen synthesis pathway, indicating ECM protein remodeling within the dermis. PM exposure caused an upregulation in *COL1A1* gene expression whereas ozone and a combination of ozone and 0.01 μg/μL PM exposure caused downregulations, indicating changes in procollagen transcript levels. Previous studies have shown PM to modulate collagen synthesis pathways after skin exposure and have shown PM to be involved in fibrosis pathways.^30^, ^31^, ^32^ Collagen‐I protein expression mirrored that of *COL1A1* gene expression, with an observed concentration‐dependent increase in collagen staining after PM exposure, specifically within the papillary dermis, and a decrease in collagen‐I staining after ozone exposure. This shows that both PM and ozone can modulate multiple points of the collagen synthesis pathway, from transcription, to procollagen folding, to translation.

The degradative action of MMPs will also contribute to collagen levels, and complimentary increases in both glycosylated and unglycosylated proMMPs were observed after exposure to ozone and a combination of ozone and 0.01 μg/μL PM. In addition to proMMP levels, the MMP activation status is also one to be considered, and while no intracellular activation was observed for MMP‐1, there was an intracellular increase in activation‐associated fragmentation of MMP‐3 after exposure to ozone and a combination of ozone and 0.01 μg/μL PM. This intracellular activation is most likely attributed to the induction of oxidative stress after ozone exposure, with ROS shown to modulate both expression and activation of MMPs.^33^ In contrast PM exposure caused decreases in MMP‐3 gene and protein expression; this could be linked to the reduction in epidermal thickness, with the lower number of viable keratinocytes either secreting less MMPs themselves, or limiting the cross talk to fibroblasts to induce expression of MMPs. With previous research showing PM to increase the expression of MMPs in dermal fibroblasts, this indicates PM has limited penetrative potential to reach the dermal layer.^34^ Coculture with keratinocytes has been shown to downregulate collagen secretion and upregulate MMP expression in fibroblasts through cytokine release, it is therefore plausible that the pollutant‐induced changes in viable epidermal thickness observed may be the cause of the changes in collagen synthesis and the MMP‐induced degradation.^35^, ^36^ MMP‐3 has the ability to not only degrade ECM proteins such as collagens II, III, and IV, but also activate other MMPs, namely MMP‐1. This was in fact observed after exposure to ozone and a combination of ozone and 0.01 μg/μL PM, which were both shown to induce an increase in activation‐associated MMP‐1 fragments in the culture medium. Since proMMPs are secreted, higher levels of activation occur extracellularly, the collagenase MMP‐1 can then degrade fibrillar type I and III collagens, with those fragments being further degraded by MMP‐3 and MMP‐9.^37^ This raises the question whether the MMP‐1 activation is induced by the pollutant‐induced MMP‐3 activation, or if the ROS generation induced by ozone mutually regulates the expression of both MMPs through MAPK activation.^38^

Lipid peroxidation was observed in skin equivalents exposed to ozone using the lipid peroxidation marker 4‐HNE, specifically in the stratum corneum and to a lesser degree in the viable epidermis. Lipid ozonation products are thought to activate phospholipases which can cause hydrolysis‐induced release of arachidonic acid which can then be further converted into multiple prostaglandin types, these prostaglandins can then mediate inflammatory response.^39^ No significant changes were observed after PM exposure. In addition to changes in lipid peroxidation status, changes in lipid synthesis status were also observed after pollutant exposure through the analysis of HMGCR. As the rate limiting enzyme in the cholesterol synthesis pathways, alteration in this enzyme level will cause changes in cholesterol synthesis, which is not only involved in the diffusion barrier of the cellular membranes, but also comprises the lipid barrier of the stratum corneum.^40^ When assessing HMGCR protein expression, the antibody recognized one band at 45 kDa, which was much lower than the predicted 100 kDa molecular weight of HMGCR. We also observed this band with an additional HMGCR antibody clone, and a similar trend was observed with *HMGCR* gene expression, indicating this band does represent HMGCR. There is very little research analyzing HMGCR protein expression in skin, but it is possible that the higher cholesterol abundance within the skin results in HMGCR existing as a lower molecular weight cleaved form natively.^41^ Other reports have indicated that bands in the 42‐62 kDa region are formed as a result of proteolysis into a C‐terminal cleavage product in the endoplasmic reticulum so the presence of this band could depend on solubilization.^42^, ^43^ Interestingly, exposure to 0.01 μg/μL PM induced an increase in HMGCR protein expression; however, 0.05 μg/μL PM induced a decrease, and ozone induced a further decrease. This concentration‐dependent effect could indicate that low concentrations of PM cause minor barrier disruption which the skin is capable of repairing by inducing upregulation of skin barrier components; however, a higher concentration of PM may overwhelm skin barrier repair systems. Some reports have shown that acute UV exposure upregulated HMGCR; however, chronic exposure downregulated HMGCR expression, so it is possible that pollutant‐induced barrier disruption follows a similar mechanism.^44^ Immunohistochemistry was also conducted to confirm the Western blot results, this showed that the expression of HMGCR within the viable epidermis mirrored the protein expression changes observed with Western blotting (data not shown).

With inflammation and wrinkling showing a tendency to be mutually induced and shown to be characteristic of a senescent phenotype, inflammatory mediator PGE2 release was investigated. Ozone exposure induced a significant increase in PGE2 secretion, and would implicate ozone in the modulation of arachidonic acid metabolism from phospholipids. As mentioned previously, lipid ozonation products are thought to activate phospholipases which can cause hydrolysis‐induced release of arachidonic acid.^39^ The oxidative stress induced by ozone can upregulate cyclooxygenase (COX) expression and PGE synthase through NF‐κB activation which can then convert arachidonic acid into prostaglandins.^45^ Although an increase in epidermal thickness will induce an overall increase in basal secretion of PGE2, this increase would be proportional to each other and the increase in PGE2 is therefore likely to be induced by pollutant exposure. However, basal secretion of PGE2 should be considered in the cases of other stressors that have been shown to increase both epidermal thickness and PGE2 secretion, such as UV.^46^ With the induction of inflammatory mediator release, it would be of interest to determine how this would influence the action of inflammatory cell if they were to be incorporated into the skin equivalents.

Synergistic properties of pollutants were also investigated, with the study being the first of its kind to do so to our knowledge. A combination of ozone and PM did not induce any significant synergistic changes in epidermal thickness/structure or PGE2 secretion when compared to ozone exposure alone; however, synergistic effects were observed with MMP‐1 and MMP‐3 gene/protein expression, and lipid peroxidation within the stratum corneum. Since ozone exerts its damage through oxidative stress, it is possible that the addition of PM could cause cumulative oxidative stress, or that the cellular ATP pool is not sufficient to facilitate adaptive mechanisms for the distinct ozone and PM‐induced damage. Since the global population are rarely exposed to just one type of air pollutant, synergistic effects should be taken into consideration when assessing pollution‐induced effects; of particular interest are air pollutant interactions with UV, as UV has been shown to photoactivate air pollutants such as PAHs and augment their damaging effects^47^

## 5 CONCLUSION

PM showed very little effect on downstream damage markers evaluated in this study, and only showed surface level effects on epidermal structure, with changes characteristic of a chloracne phenotype. By contrast, ozone was shown to have an effect on both epidermal structure and downstream markers, inducing upregulations in the gene/protein expression of wrinkling‐associated MMP‐1 and MMP‐3, and also the release of inflammatory mediator PGE2. Although PM alone was not damaging enough to alter any downstream markers, when combined with ozone there were significant synergistic effects observed, resulting in acceleration of the aging phenotype in skin equivalents.

This study identifies some of the distinct effects of various air pollutants, and how exposure to multiple pollutants may enhance aging‐associates changes; these factors should be considered during investigation into the effects of air pollution and during protective compound screening.

## AUTHOR CONTRIBUTIONS

M.A. Birch‐Machin as the corresponding author codesigned the research proposal with B. Chavan. W.J. Reynolds performed the experiments and wrote the paper. P. Christensen and N. Eje for developing the ozone generation system. M.A. Birch‐Machin, B. Chavan, W.H. Li, S.M. Daly, and R. Parsa provided comments on the manuscript.

## CONFLICT OF INTEREST STATEMENT

W.H. Li, S.M. Daly, and R. Parsa were employees of Johnson and Johnson Consumer Inc. when these experiments were conducted. The other authors declare no conflict of interest.

## Supporting information


Figure S1

## Data Availability

The data that support the findings of this study are available from the corresponding author upon reasonable request.
