# Supplementary material for: Biological effects of air pollution on the function of human skin equivalents
Source: FASEB Bioadv. 2023 Oct 3;5(11):470–83. doi: 10.1096/fba.2023-00068 (PMC10626160; doi:10.1096/fba.2023-00068)
Supplement: Supplementary file 1 — Figure S1 [file FBA2-5-470-s001.zip › fba21412-sup-0002-Supinfo.pdf]

Supplementary Figure 1: Secondary-only immunohistochemical controls.
Phenion FT skin equivalent sections were blocked, and incubated with ImmPRESS Universal Antibody Polymer Reagent for chromogenic immunohistochemistry (A), or anti-mouse Alexa Fluor 594 secondary antibody for fluorescent immunohistochemistry (B), in the absence of any primary antibody to determine any non-specific secondary binding or autofluorescence. Cell nuclei were counterstained with haematoxylin (A) or with DAPI (B). Scale bar: 50µm.
