# Supplementary figures and images for: Biological effects of air pollution on the function of human skin equivalents
Source: FASEB Bioadv. 2023 Oct 3;5(11):470–83. doi: 10.1096/fba.2023-00068 (PMC10626160; doi:10.1096/fba.2023-00068)

**A**

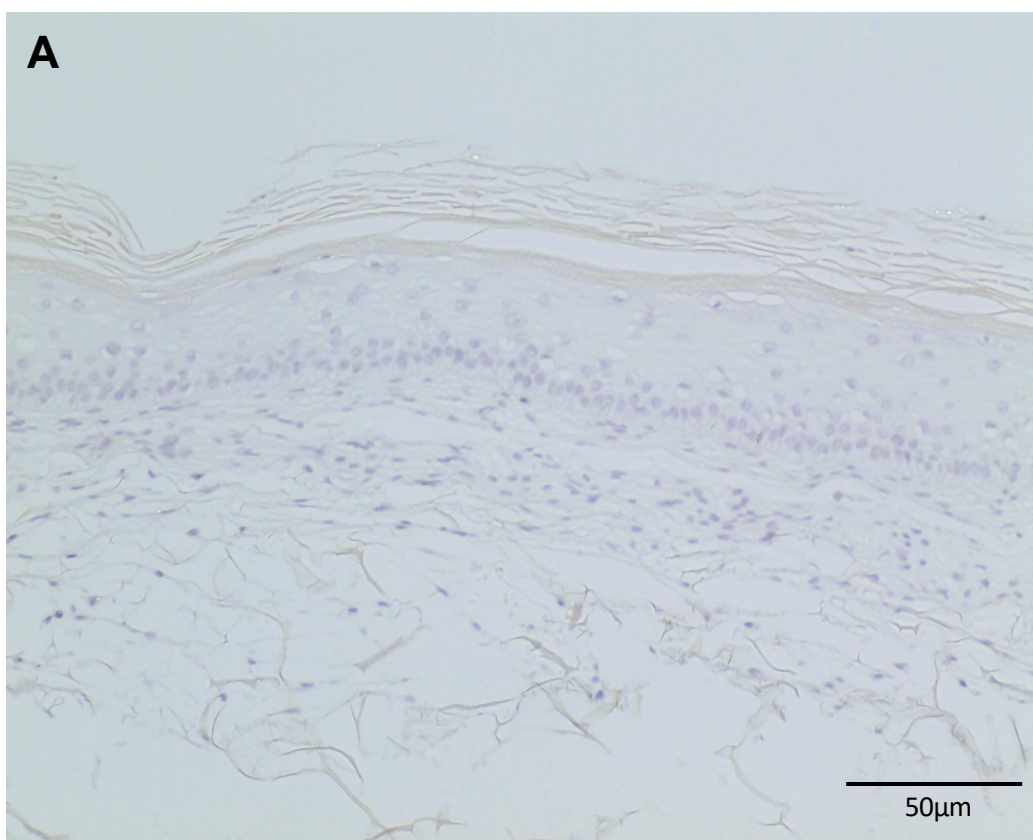

**B**

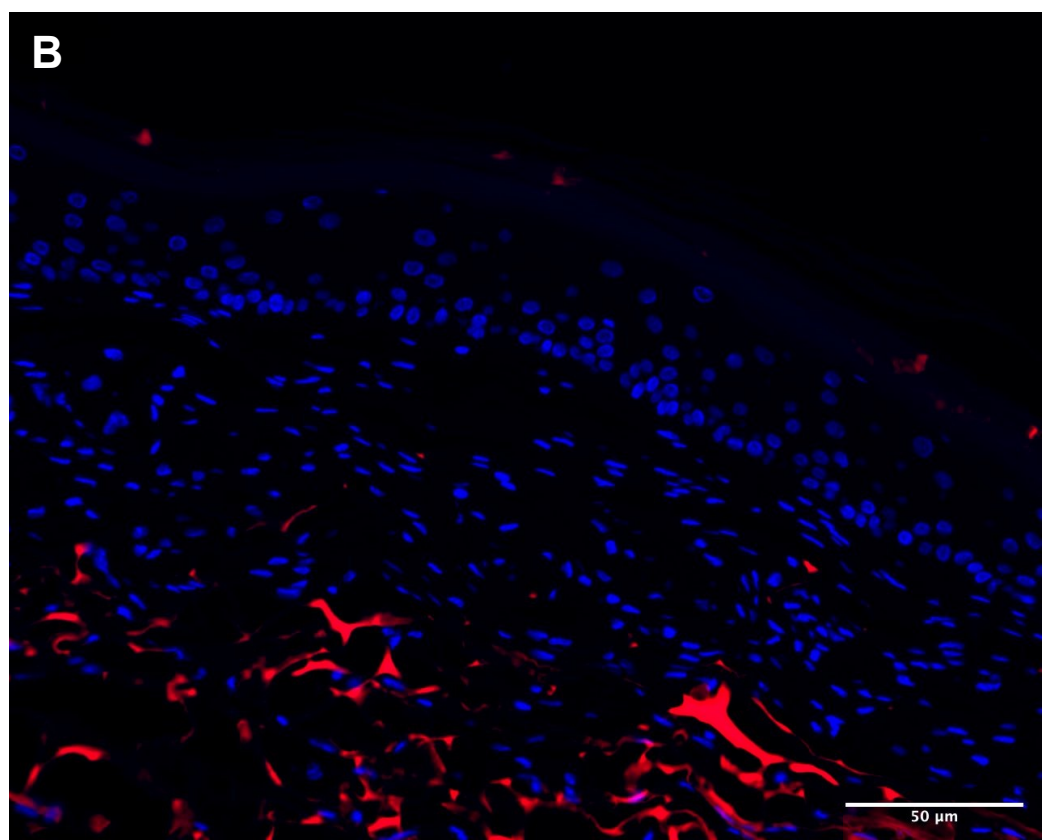

Supplement: Supplementary file 1 — Figure S1 [file FBA2-5-470-s001.zip › fba21412-sup-0001-FigureS1.pdf]
